# Multiplexed Imaging Strategy to Distinguish Indeterminant Biliary Strictures: An *Ex Vivo* Study

**DOI:** 10.47690/wjghe.2020.3303

**Published:** 2020-10-08

**Authors:** MB Sturm, BP Joshi, SR Owens, EJ Seibel, TD Wang

**Affiliations:** 1 Department of Internal Medicine, Division of Gastroenterology, University of Michigan, Ann Arbor, MI, 48109, USA; 2 Department of Pathology, University of Michigan, Ann Arbor, 48109, USA; 3 Department of Mechanical Engineering, University of Washington, Seattle, WA 98195, USA; 4 Department of Biomedical Engineering, University of Michigan, Ann Arbor, MI, 48109; 5 Department of Mechanical Engineering, University of Michigan, Ann Arbor, MI, 48109

**Keywords:** Cholangiocarcinoma, Biliary Neoplasia, Fluorophores, Immunofluorescence, Oncoprotein Peptide Labeling

## Abstract

**Introduction:**

Indeterminant biliary strictures can be either malignant or benign. Biliary intraepithelial neoplasia (BilIN) is the precursor lesion to cholangiocarcinoma, a deadly bile duct cancer. Current diagnostic methods are limited by inadequate amounts of cells and tissues collected.

**Aim:**

We aim to demonstrate use of fluorescently-labeled peptides specific for EGFR, claudin-1, and ErbB2 to perform multiplexed imaging of biliary neoplasia.

**Methods:**

Formalin fixed and paraffin embedded specimens resected from human biliary strictures were sectioned. A gastrointestinal pathologist used standard criteria to score immunohistochemistry from biliary neoplasia and adjacent normal epithelium from the same specimen. Peptides specific for EGFR, claudin-1, and ErbB2 were fluorescently-labeled with FITC, Cy5, and IRDye800, respectively. The fluorophores were chosen to provide spectral separation to distinguish the individual targets. Immuno fluorescence images were collected using confocal microscopy.

**Results:**

Target expression was validated using immunohistochemistry. Staining was visualized on the surface of biliary duct epithelial cells and not in the stroma. Greater fluorescence intensity was observed for peptide binding to biliary neoplasia by comparison with normal. The mean ratio for neoplasia-to-normal was 1.4, 1.7, and 1.6, respectively, and the average intensities were significantly greater for neoplasia than normal for each peptide. Peptides and antibody binding co-localized with correlation of ρ=0.64, 0.51 and 0.62, respectively.

**Conclusions:**

A panel of fluorescently-labeled peptides can distinguish BilIN and cholangiocarcinoma from normal biliary epithelium, and may be used for multiplexed imaging of indeterminant biliary strictures.

## INTRODUCTION

Cholangiocarcinoma is a primary malignancy of the biliary tract [1,2]. The incidence and mortality of this disease are increasing steadily [3]. This cancer is often diagnosed at an advanced stage when the 5-year survival rate is low and prognosis is poor [4]. Biliary intraepithelial neoplasia (BilIN) represents a precursor condition [5], and if detected early, patients can undergo surgical resection with excellent outcomes that are comparable with liver transplantation [6]. This cancer results from chronic inflammation of the bile ducts and arises from a variety of etiologies [7]. Patients suspected of having cholangiocarcinoma frequently present with indeterminant biliary strictures seen on trans abdominal imaging [8]. Surgery is the definitive therapy for this condition, but up to one quarter of resections for suspected malignant strictures actually turn out to be benign [9]. Because biliary ducts are small in caliber, only scant amounts of cells and tissues can be collected for either cytology or histology. The amounts are usually insufficient to make a definitive diagnosis. New methods of imaging can help to accurately identify cholangiocarcinoma in indeterminant biliary strictures to guide physicians in making the most appropriate therapeutic decision.

Because cholangiocarcinoma is a heterogeneous disease, a single target is unlikely to be adequate for the general patient population [10,11]. An imaging approach that can detect multiple targets at the same time (multiplexing) may achieve high diagnostic performance. Light has a broad spectrum that can be separated into several channels to image multiple targets concurrently. Flexible fiber endoscope accessories are being developed with sufficiently small dimensions to pass easily through side viewing medical endoscopes into the biliary ducts [12]. Peptides can be developed to bind with high affinity to cell surface targets that are over expressed in neoplasia by comparison with normal epithelium and are accessible to imaging [13–15]. They can be labeled with a wide range of fluorophores and can bind rapidly *in vivo* with topical application [16,17]. These imaging agents provide a biological basis for detecting disease, establishing prognosis, guiding therapy, and monitoring treatment. Methods of targeted imaging using bright fluorophores can highlight areas of neoplasia to guide either biopsy or surgery of biliary tract cancers. The pathogenesis of cholangiocarcinoma is heterogeneous [18–20].

A stepwise transformation from normal biliary epithelium into cholangiocarcinoma results from an accumulation of diverse molecular changes. The genetic pathways that contribute to the selective growth advantage of these cancer cells are being elucidated. EGFR and ErbB2 (HER2) are receptor tyrosine kinases that have been identified on comprehensive genomic profiles to be over expressed in cholangiocarcinoma [21,22]. These findings have been validated using immunohistochemistry. Claudins are a family of trans membrane proteins that serve as integral components of tight junctions in the biliary duct epithelium, and are known to be highly expressed in a number of epithelial cancers [23]. However, the molecular targets for BilINs have not been elucidated. Here, we hypothesized that EGFR, claudin-1, and ErbB2 are over expressed on the cell surface in BilINs, the precursor lesion, and in cholangiocarcinoma, and can serve as targets for multiplexed imaging for future *in vivo* imaging to distinguish indeterminant biliary strictures.

## RESULTS

### Immunohistochemistry

**Figure 1:**
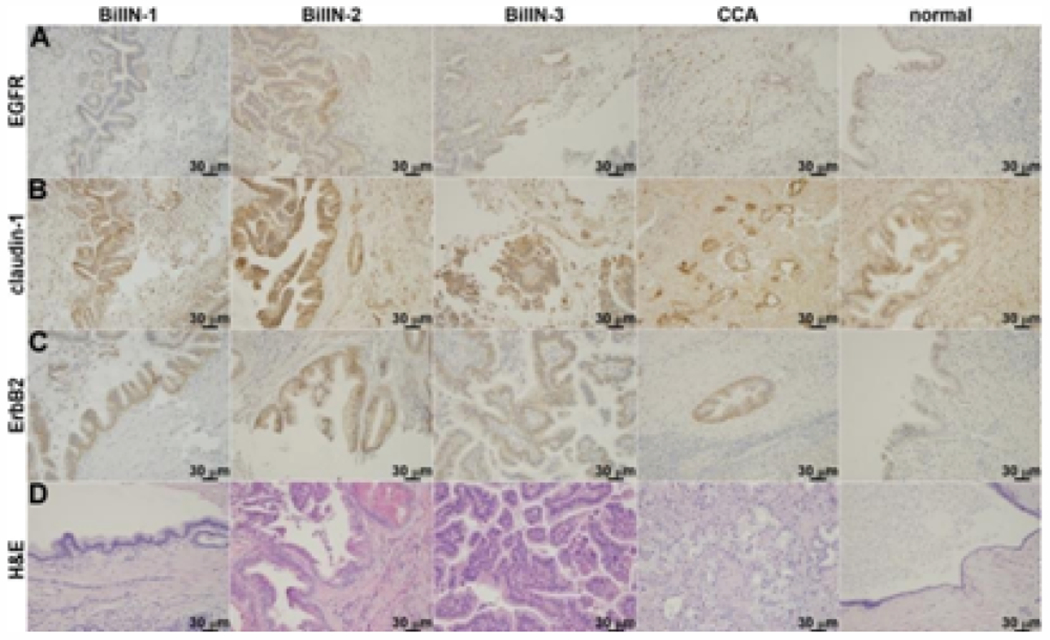
Immunohistochemistry. Representative sections of resected human indeterminant biliary stricture specimens were stained for expression of **A)** EGFR, **B)** claudin-1, and **C)** ErbB2. Increased reactivity is seen in human BilIN-1, -2, -3, and cholangiocarcinoma (CCA) by comparison with normal biliary epithelium. **D)** Representative histology (H&E) is shown for each classification.

Immunohistochemistry (IHC) was performed in n = 24 formalin-fixed, paraffin-embedded (FFPE) sections from specimens of resected human indeterminant biliary strictures to evaluate the expression of EGFR, claudin-1, and ErbB2, (Figure 1A–C). Strong staining (2+/3+) was observed for BilIN-1, BilIN-2, BilIN-3, and cholangiocarcinoma (CCA). Increased reactivity was seen in ductal epithelial cells by comparison with the stroma. Normal biliary epithelium was identified in n=21 specimens, and weak staining (0+/1+) was observed. Representative histology (H&E) is shown for each classification of pathology (Figure 1D). Biliary intraepithelial neoplasia (BilIN) was found in n=21 specimens, including BilIN-1 (n=7), BilIN-2 (n=4), BiliN-3 (n=6), and mixed (n = 4) (Table 1). At least one identifiable focus of CCA, including intrahepatic (n=6), perihilar (n=3), extrahepatic (n=6), and unknown (n=1), was seen in n = 16 specimens. BilIN pathology was frequently seen adjacent to invasive cancer. All samples contained some evidence of inflammation.

### Fluorescently-labeled Peptides

**Figure 2:**
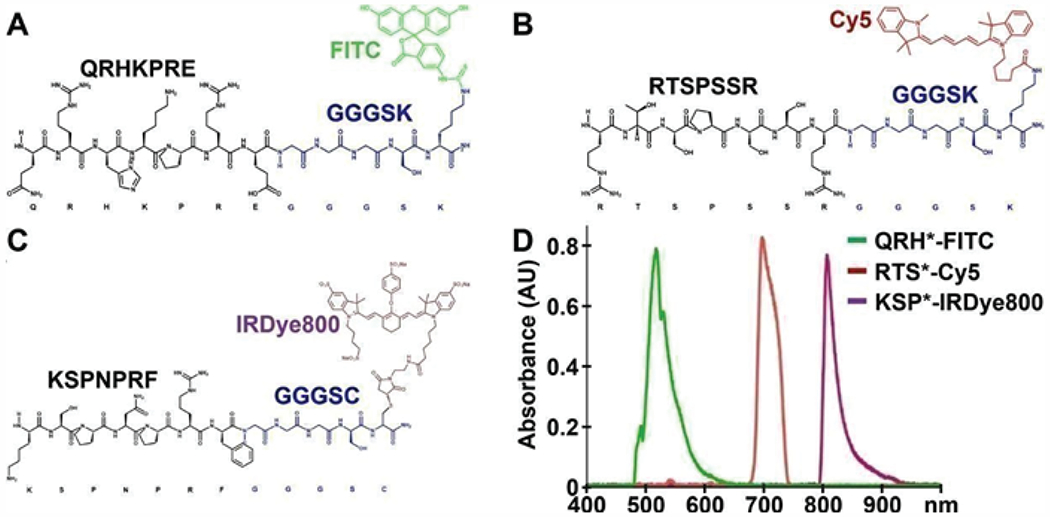
Peptide imaging agents. Biochemical structures are shown for peptide sequences (black) specific for **A)** EGFR, **B)** claudin-1, and **C)** ErbB2 with linkers (blue) to attach fluorophores FITC, Cy5, and IRDye800, respectively. **D)** Fluorescence spectra with excitation at λex = 488, 660, and 785 nm resulted in emission with peaks at λem = 519, 699, and 808 nm, respectively, and showed minimal overlap.

A panel of fluorescently-labeled peptides was developed to perform multiplexed detection of EGFR, claudin-1, and ErbB2. The peptide QRHKPRE specific for EGFR was labeled with FITC via a GGGSK linker to prevent sterichindrance, hereafter QRH* FITC (Figure 2A) [13]. RTSPSSR, specific for claudin-1, was labeled with Cy5 via a GGGSK linker, hereafter RTS*-Cy5, (Figure 2B) [14]. KSPNPRF, specific for ErbB2, was labeled with IRDye800 via a GGGSC linker, hereafter KSP*-IRDye800 (Figure 2C) [15]. The fluorophores FITC, Cy5, and IRDye800 were chosen to span the visible and near-infrared spectrum. The fluorescence emission spectra of this panel of peptides showed minimal overlap (Figure 2D).

### Immunofluorescence

**Figure 3:**
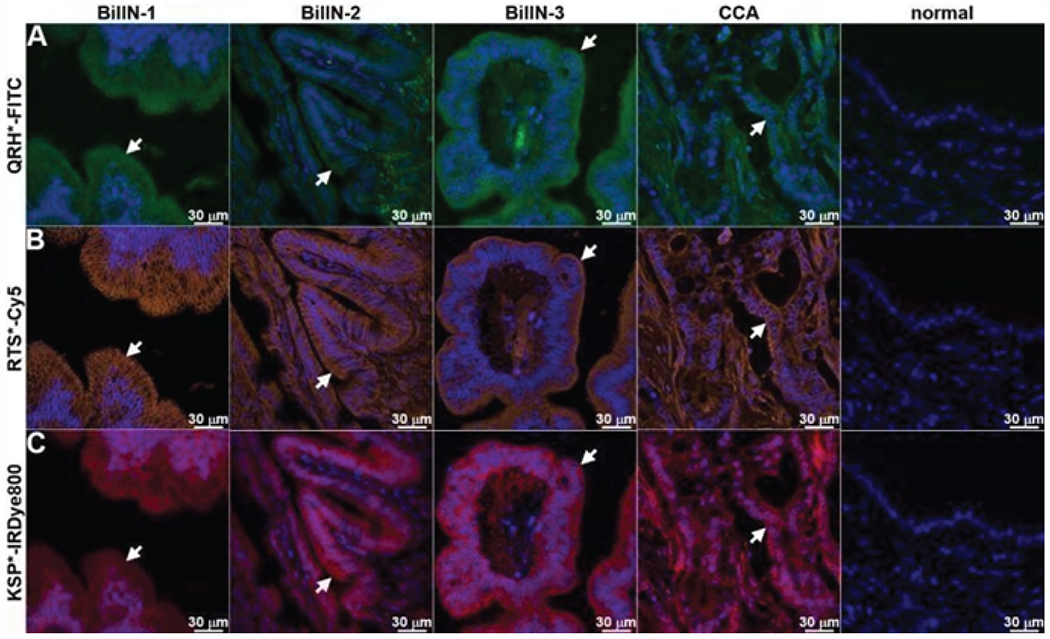
Immunofluorescence. Using QRH*-FITC, RTS*-Cy5, and KSP*-IRDye800, bright signal is seen at the cell surface (arrows) for BilIN-1, -2, -3, and cholangiocarcinoma (CCA) while minimal intensity is seen in the stroma. By comparison, minimal signal is seen in normal biliary duct epithelium.

**Figure 4:**
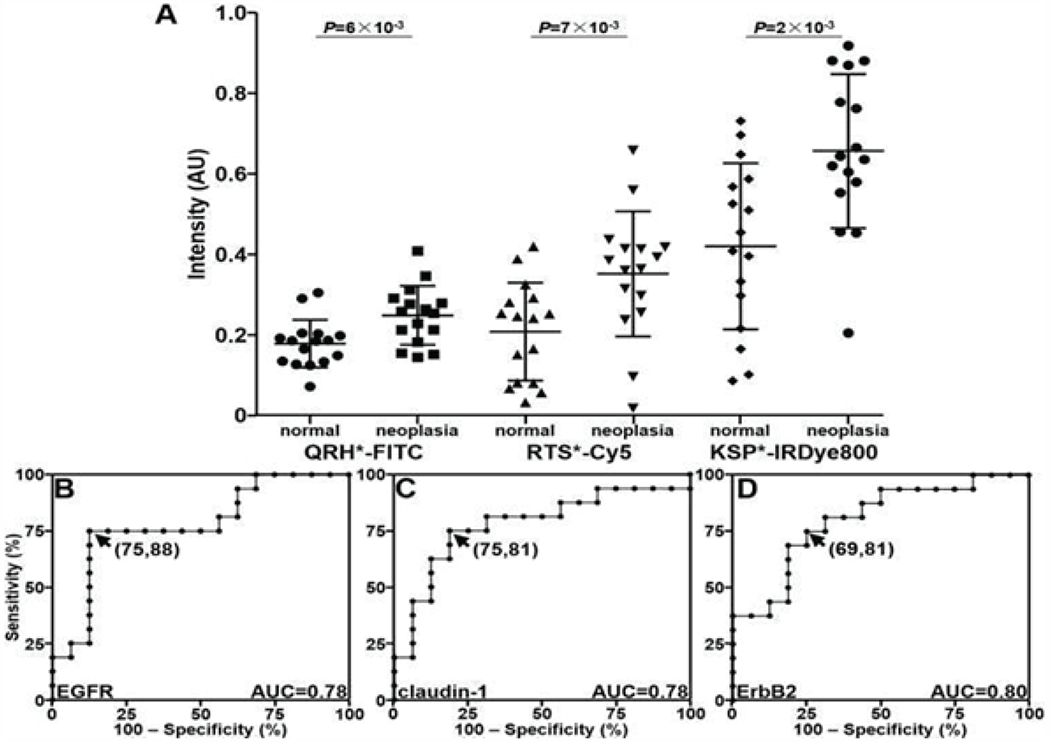
Quantified fluorescence intensities. **A)** Scatter plot shows individual values for specimens of biliary neoplasia, including BilIN-1, BilIN-2, BilIN-3, and CCA, compared with that for normal epithelium. Differences in mean results are significant for QRH*-FITC (EGFR), RTS*-Cy5 (claudin-1), and KSP*-IRDye800 (ErbB2) with P = 6×10-3, 7×10-3, and 2×10-3 by unpaired t-test, respectively. ROC curves show optimal sensitivity and specificity of **B)** 75% and 88% for EGFR, **C)** 75% and 81% for claudin-1, and **D)** 69% and 81% for ErbB2 with AUC of 0.78, 0.78, and 0.80, respectively.

QRH*-FITC, RTS*-Cy5, and KSP*-IRDye800 were applied to the biliary specimens and imaged using confocal microscopy. Greater fluorescence intensity was observed from binding of each peptide to the surface (arrows) of ductal epithelial cells in specimens of biliary neoplasia, including BilIN-1, BilIN-2, BilIN-3, and CCA, by comparison with that for normal (Figure 3A–C). The fluorescence intensities were quantified, and the mean ratios for neoplasia to normal was 1.4, 1.7, and 1.6, respectively. The mean values were found to be significantly greater for neoplasia than that for normal for each peptide (Figure 4A). ROC curves showed 75%, 75%, and 69% sensitivity and 88%, 81%, and 81% specificity for distinguishing neoplasia from normal for EGFR, claudin-1, and ErbB2, respectively (Figure 4B–D). An AUC of 0.78, 0.78, and 0.80, respectively, were found. Combining the peptidesas a panel resulted in 94% sensitivity and 69% specificity for detection of biliary neoplasia.

### Co-localization of antibody and peptide binding

**Figure 5:**
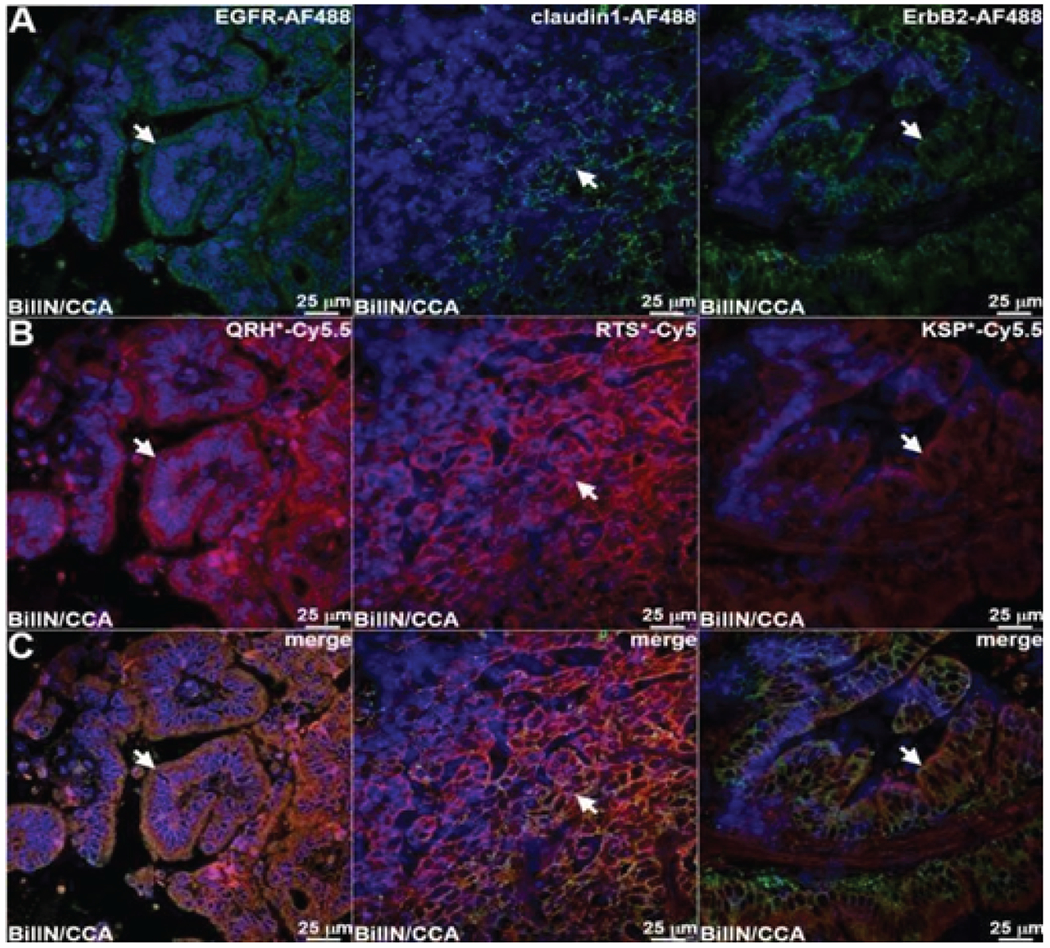
Co-localization of antibody and peptide binding to biliary neoplasia. **A)** Fluorescence images collected with confocal microscopy show AF488-labeled antibodies (green) specific for EGFR, claudin-1, and ErbB2 binding to the cell surface (arrows) of neoplastic biliary epithelial cells. **B)** Peptides labeled with either Cy5.5 or Cy5 (red) also show cell surface binding (arrows) to adjacent sections. **C)** Merged images show co-localization of binding (arrows) with a Pearson’s correlation coefficient of ρ = 0.64, 0.51 and 0.62 for EGFR, claudin-1, and ErbB2 respectively.

**Figure 6:**
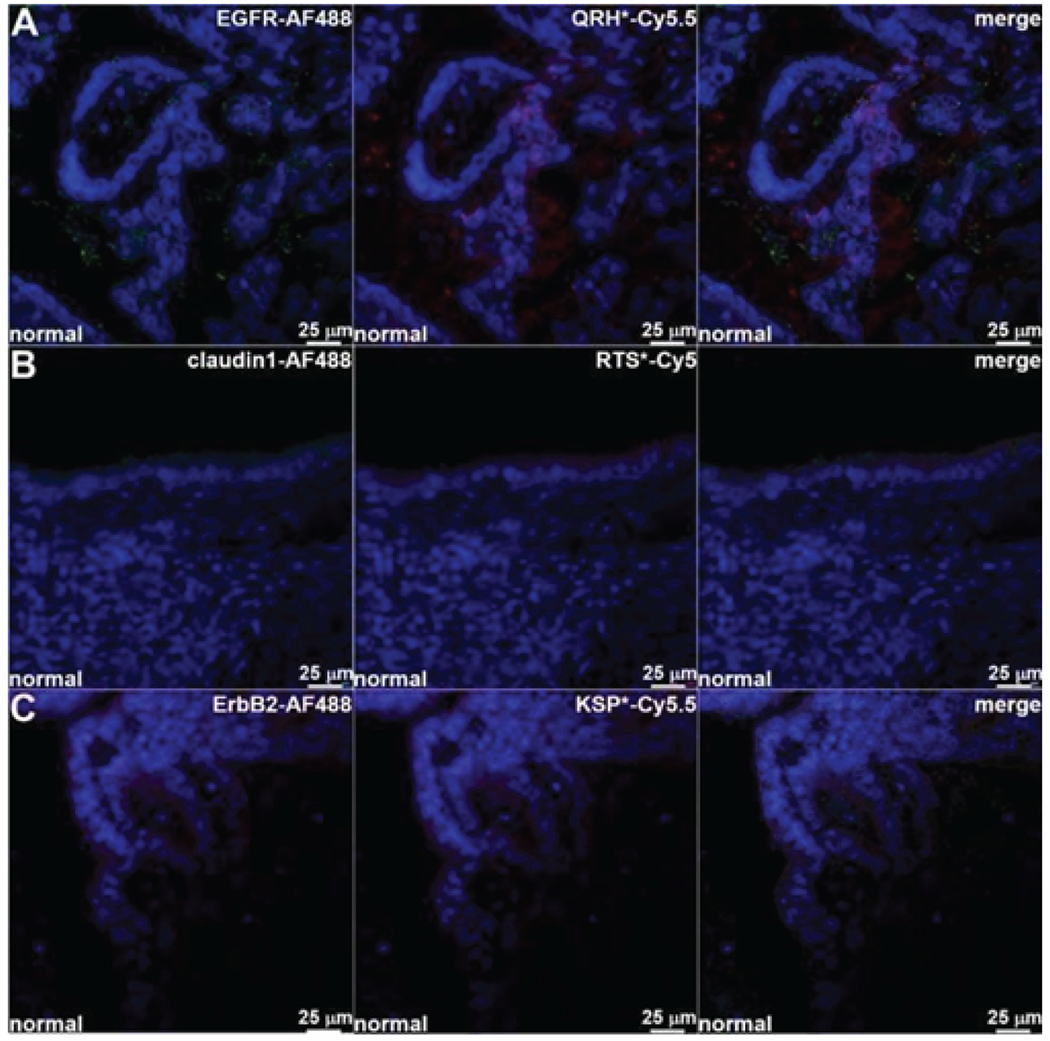
Co-localization of antibody and peptide binding to normal biliary epithelium. Fluorescence images collected with confocal microscopy show minimal binding of AF488-labeled antibodies and Cy5.5/Cy5-labeled peptide specific for **A)** EGFR, **B)** claudin-1, and **C)** ErbB2.

Known antibodies specific for EGFR, claudin-1, and ErbB2 were labeled with AF488, and the peptide panel was labeled with either Cy5.5 or Cy5. Both antibody and peptide binding to the surface (arrows) of neoplastic biliary epithelial cells is seen on fluorescence images collected with confocal microscopy (Figure 5A–C). Merged fluorescence images showed strong co-localization of peptide and antibody binding for each target. A Pearson’s correlation coefficient of ρ = 0.64, 0.51 and 0.62 was determined for EGFR, claudin-1, and ErbB2, respectively. By comparison, minimal binding with either peptide or antibody was seen for normal biliary epithelium (Figure 6A–C).

## METHODS

### Immunohistochemistry

Formalin-fixed and paraffin-embedded (FFPE) specimens resected from human indeterminant biliary strictures were obtained from the archived tissue bank in the Department of Pathology at the University of Michigan. The specimens were cut in 5 μm sections, mounted onto glass slides (Super frost Plus, Fischer Scientific) and heat fixed. The tissues were then deparaffinized by incubating the slides 3X in xylene for 5 min. Hydration was performed by washing the slides 2X in 100% ethanol for 2 min, 2X in 95% ethanol for 2 min, and 2X in distilled H_2_O (dH_2_O) for 3 min. Antigen unmasking was performed by boiling the slides in citrate buffer (10 mM citric acid, 0.05% Tween-20, pH 6.0), maintaining a sub-boiling temperature for 15 min, then gradual cooling at room temperature (RT) for 30 min. The sections were washed 3X in dH_2_O for 3 min. Endogenous peroxidase activity was inactivated via incubation with 3% hydrogen peroxide/methanol for 10 min. Sections were then washed 3X in dH2O for 2 min and placed in phosphate buffered saline (PBS) with 0.1% Tween-20 (PBST). A serum free protein blocking solution (#X0909, Dako) was applied for 30 min at RT. Sections were then washed 1X in PBS. Primary antibodies, including EGFR (clone 111.6, Thermo Scientific,) claudin-1 (#ab15098, Abcam), and ErbB2 (#2165, Cell Signaling Technology), respectively, were incubated at dilutions of 1:400, 1:200, and 1:400. The slides were placed in antibody diluent buffer (#S0809, Dako) overnight at 4°C in a humidified chamber. Sections were then washed 3X in PBST for 5 min. A 1:200 dilution of biotinylated secondary antibody (goat anti-rabbit or anti-mouse IgG) was incubated with the sections in antibody diluents buffer for 30 min at RT and then washed 3X in PBST.

Elite Vectastain ABC reagent (Vector Labs) was added to each section and incubated for 30 min at RT. The sections were washed 3X in PBS for 5 min, and developed with 3,3′-diaminobenzidine tetrahydrochloride substrate (Sigma-Aldrich). The reaction was monitored for up to 10 min, and quenched by immersing the slides in dH_2_O. Hematoxylin was added as a counterstain for approximately 20 sec, and the sections were dehydrated in increasing concentrations of ethyl alcohol (2X at 70%, 80%, 95%, and 100%). Coverslips were mounted using per mount mounting medium (#SP15-100; Fisher) in xylene. Serial sections were processed for routine histology (H&E).

A gastrointestinal pathologist (SO) reviewed the IHC sections and assigned grades for BilIN based on previously reported histopathological criteria [24]. Scoring was performed using a standard system. Each section was assigned to the highest grade of BilIN as being either positive (2/3+) or negative (0/1+). Adjacent normal biliary epithelium from the same specimen was used as control.

### Fluorescently-labeled peptides

The fluorescently-labeled peptides were synthesized using standard solid phase Fmoc chemistry, as described previously [25]. 5-FITC was obtained from Sigma Aldrich. Cy5 and Cy5.5 NHS ester were obtained from Lumiprobe. IRDye800CW maleimide was obtained from LI-COR Biosciences. Fluorescence emission was collected with a fiber coupled spectrophotometer (Ocean Optics) using excitation at λex =488, 660, and 785 nm. The spectra were plotted using Origin 6.1 software (Origin Lab Corp).

### Immunofluorescence

For multiplexed imaging, the tissue sections were deparaffinized and antigen unmasked, as described above. The slides were then washed 3X in dH_2_O for 2 min each and placed in PBS. Blocking was performed, as described above. A solution containing 2.5 μM of each peptide in PBS was incubated on each slide for 5 min and washed 3X for 5 min in PBS. Coverslips were mounted with Prolong Gold with DAPI (Invitrogen) as an anti-fade reagent. Confocal fluorescence images were collected with an Olympus FV1200 microscope using a 20X water immersion objective. The laser power, detector gain, and image acquisition times were kept constant for all images. The fluorescence intensities were quantified by measuring the mean fluorescence intensity from 4 solid boxes with dimensions of 30×30 μm^2^ that contained biliary epithelium. Regions of saturated image intensities were avoided. Image J software was used. The target-to-background ratios were determined by ratioing the mean intensities from biliary neoplasia and adjacent normal epithelium.

### Co-localization of antibody and peptide binding

Formalin-fixed and paraffin-embedded (FFPE) specimens resected from human biliary strictures were sectioned. The slides were hydrated, and antigen retrieval was performed, as described above. After citrate antigen retrieval and blocking, 2.5 μM of either QRH*-Cy5.5, RTS*-Cy5, or KSP*-Cy5.5 was incubated on slides for 5 min at RT. The slides were washed 1X with PBS and incubated with primary antibodies at 4°C overnight in a humidified chamber per manufacturer recommendations. The slides were then washed 4X in PBS. Secondary AF488-labeled antibody was incubated at RT for 30 min, and washed 3X for 5 min in PBS. Coverslips were mounted with anti-fade reagent Prolong Gold with DAPI (Invitrogen). Confocal imaging was performed using DAPI, AF488, and Cy5/Cy5.5 filter sets. Co-localization of peptide and antibody binding was evaluated by calculating the Pearson’s correlation coefficient.

## DISCUSSION

Here, we demonstrate evidence to support the use of a panel of fluorescently labeled peptides specific for EGFR, claudin-1, and ErbB2 to distinguish BilIN, the precursor lesion, and cholangiocarcinoma from normal biliary epithelium. These targets arise from different molecular processes, and were chosen to address the heterogeneity of this disease. Target expression was visualized on the cell surface of the biliary ducts, thus are amenable for future *in vivo* imaging. These fluorophores provide sufficient spectral separation to distinguish the individual targets. Specimens obtained from resected human indeterminant biliary strictures were evaluated. Heterogeneous expression of this panel of targets was validated using immunohistochemistry. Histology of the regions imaged was confirmed by an expert pathologist. The target panel was found to be over expressed in all grades of BilIN, and biliary neoplasia was identified with high sensitivity and specificity.

These results suggest that multiplexed imaging using a peptide panel is promising for clarifying indeterminant biliary strictures. Indeterminate biliary strictures are non-specific clinical findings seen on cross-sectional imaging, including ultrasound, CT, and MRI. This condition can arise from either benign or malignant lesions, and accurate diagnosis is critical to avoid unnecessary surgery. Patients identified at an early stage are eligible for surgical resection and may have excellent outcomes [6]. Unfortunately, up to 25% of patients with this condition undergo surgery for presumed cancer but are found afterwards to have a benign condition instead [9]. Minimally invasive evaluation with endoscopic retrograde cholangiopancreatography (ERCP) with brush cytology frequently results in an inadequate number of cells obtained [26]. Also, endoscopic ultrasound (EUS) with fine needle aspiration (FNA) often provides a limited amount of tissue, and risks seeding of malignant cells thus eliminating patient eligibility for transplantation [27]. Serum biomarkers, such as CA 19-9 and CEA, have not been found to be sufficiently specific to be used routinely [28]. Adjunct molecular techniques, such as fluorescence *in situ* hybridization (FISH), have not shown a significant improvement in performance [29].

New methods of imaging may improve the diagnostic accuracy for this condition. Multiplexed detection of a panel of fluorescently-labeled peptides has been previously demonstrated *in vivo* in a preclinical model of epithelial cancer [30]. These peptides were identified empirically and labeled with a set of visible fluorophores. A rigid small animal endoscope was used to collect fluorescence images in real time. Multispectral detection was performed using a unique set of lasers to deliver 3 excitation wavelengths. An optical system consisting of dichroic mirrors and filters was used to separate the fluorescence collected into separate channels. The visible and NIR fluorophores used in this study, including FITC, Cy5 and IRDye800, provide adequate spectral separation to clearly distinguish the 3 individual targets. Topical administration of fluorescently-labeled peptides has been previously demonstrated clinically in esophagus and colon. This approach delivers the peptides to epithelial surfaces effectively in high concentrations to maximize target interactions and achieve rapid binding with minimal risk for toxicity.

While these results demonstrate promise for use of multiplexed imaging methods to distinguish indeterminant biliary strictures, the conclusions reached are limited by the *ex vivo* nature of the study performed. While use of the EGFR and claudin-1 peptides together showed comparable performance to the complete peptide panel, ErbB2 was expressed in some specimens that lacked either target. Fluorescence intensities from biliary neoplasia was compared with that from normal epithelium. Specimens with varying degrees of inflammation would better control the study. A flexible fiber instrument that can be passed through the working channel of a side viewing medical endoscope is needed, and the optical detection scheme should be assembled into a portable system. In this study, specimens from intrahepatic, perihilar, and extrahepatic lesions were combined. The results may differ for the different an atomic subtypes given the broad etiologies that drive this disease. Furthermore, evaluation of other targets may improve diagnostic performance.

## Figures and Tables

**Table 1:** Immunohistochemistry. Expression of EGFR, claudin-1, and ErbB2 in n = 24 resected human biliary stricture specimens was evaluated using immunohistochemistry. Staining results for normal, BilIN-1, BilIN-2, BilIN-3, and cholangiocarcinoma (CCA) are shown. Mixed grades included BilIN-1/3 (n = 2) and BilIN-2/3 (n =2).

Target	EGFR	claudln-1	ErbB2
**Total (n=24)**	7+/14−	20+/1−	7+/14−
**BILIN-1 (n=7)**	3+/4−	6+/1−	6+/1−
**BILIN-2 (n=4)**	1+/3−	3+/1−	3+/1−
**BILIN-3 (n=6)**	2+/5−	6+/0−	5+/1−
**mixed (n=4)**	1+/3−	3+/1−	4+/0−
**normal (n=21)**	0+/21−	5+/16−	1+/20−
**CCA (n=16)**	6+/10−	15+/1−	10+/6−

## ABBREVIATIONS

BilIN: Biliary Intraepithelial Neoplasia
CCA: Cholangiocarcinoma
EGFR: Epidermal Growth Factor Receptor
ERCP: Endoscopic Retrograde Cholangiopancreatography
EUS: Endoscopic Ultrasound
HER2: Human Epidermal Growth Factor Receptor 2
FITC: Fluorescein Isothiocyanate
IHC: Immunohistochemistry
IF: Immunofluorescence
